# Non-surgical endodontics - obturation

**DOI:** 10.1038/s41415-025-8562-1

**Published:** 2025-04-11

**Authors:** Carol Tait, Josette Camilleri, Katherine Blundell

**Affiliations:** 233134695108334186281 Dundee Hospital and Research School, University of Dundee, Dundee, United Kingdom; 702739096838545205586 Dentistry, School of Health Sciences, College of Medicine and Health, University of Birmingham, Birmingham, United Kingdom; 872315182059271439104 School of Dentistry, Institute of Life Course and Medical Sciences, Faculty of Health and Life Sciences, Liverpool, United Kingdom

## Abstract

Once the root canal space is shaped and disinfected, it must be sealed to prevent recontamination, which can compromise treatment success and lead to reinfection. This crucial phase of root canal therapy is known as obturation - a term derived from the Latin *obturare*, meaning to block, close, or obstruct. The primary goal of obturation is to create a bacteria-tight/fluid-tight seal within the root canal system, preventing bacterial ingress and entombing any residual microorganisms that may remain after cleaning and shaping. Achieving an optimal seal requires a thorough understanding of the various materials and techniques available, as well as their respective advantages and limitations. This paper provides a comprehensive overview of the materials and clinical techniques used in root canal obturation, including traditional and contemporary approaches. Additionally, it explores the rationale behind material selection and technique adaptation, equipping clinicians with the knowledge to make informed decisions tailored to individual clinical scenarios. By evaluating the properties and performance of different sealers and obturation methods, this paper aims to guide practitioners toward the most effective and predictable outcomes in endodontic treatment.

## Key points

- The aim of obturation is to create a bacteria- and fluid-tight seal within the root canal system, preventing reinfection and entombing any residual microorganisms remaining after cleaning and shaping.
- The paper reviews both traditional and contemporary obturation materials and techniques, outlining their respective advantages and limitations to support optimal sealing.
- Highlights the importance of understanding the properties of materials and adapting techniques to individual clinical situations, enabling informed decision-making for more predictable and successful endodontic outcomes.

## Introduction

Endodontic treatment, or root canal treatment, is necessary when the dental pulp is irreversibly damaged or undergoes necrosis and the resultant inflamed or infected (necrotic) tissue needs to be removed to treat the inflammatory and infective process. Endodontic disease is caused by noxious stimuli, such as the bacterial byproducts arising from dental caries, dental trauma, tooth wear and tooth restoration. Endodontic treatment involves the removal of the dental pulp or pulpal remnants, chemo-mechanical preparation of the root canal space, followed by root canal filling. The aim of obturation is to create a fluid-tight seal within the root canal system, preventing bacterial ingress and entombing any residual microorganisms that may remain after cleaning and shaping, to prevent persistent infection or reinfection of the root canal space.

The clinical procedure involves the mechanical preparation of the root canal space by a series of instruments that shape the root canal to achieve a continuously tapering form. This process, when accompanied by irrigation using antimicrobial chemicals such as sodium hypochlorite, facilitates a reduction of the microbial load within the root canal space. Other solutions, such as ethylenediaminetetraacetic acid, remove the smear layer which the mechanical debridement leaves behind. The endodontic procedure can be undertaken in a single or in multiple visits depending on the complexity of the root canal anatomy, the degree of infection present, and also the level of clinician's technical skill or experience. A root canal filling is undertaken once the signs and symptoms of infection have subsided. The subsequent restoration, which facilitates a coronal seal, completes the patient management and is considered to be as important in relation to success as the obturation itself. If the coronal seal is inadequate or lost over time because of tooth fracture, caries or trauma, this may result in either persistent or emergent endodontic disease developing, where the initial treatment may have been considered to be successful.^1^

## Objectives of obturation

The earliest documented root canal filling was described in 1728 by Pierre Fauchard who filled canals with lead. Several other historical materials were also reported, and gutta-percha (GP) was first introduced by Bowman in 1867.^2^

Historically, numerous techniques, mainly using GP in combination with a sealer cement, have been described to attempt to create a seal which prevents the movement of fluid or bacteria. Although root canal obturation is considered an essential step of root canal treatment, it will only be effective if root canal disinfection in combination with adequate canal preparation has achieved a satisfactory reduction of the microbial load in the root canal system. Mechanical instrumentation has been shown to only reach a proportion of the canal system, with 35-53% of the root canal system remaining un-instrumented by mechanical techniques alone.^3^ It is widely accepted that planktonic microorganisms can be effectively removed by irrigation of the root canal space using a size 27- or 30-gauge irrigation needle.^4^^,^^5^ The biofilm on the canal walls and microorganisms located within isthmuses, lateral canals and apical deltae can prove more challenging. Creating a seal apically, laterally and coronally (known as a 3D seal) is therefore required to deprive any residual organisms and their toxins access to the periapical tissues and create a biological environment which is unfavourable for bacterial growth and survival, which favours periapical healing.^6^ In this respect, root canal filling plays an essential role at the conclusion of the endodontic treatment.

## Ideal properties of an obturating material

Grossman^7^ described the features of an ideal obturating material (Box 1). None of the materials available clinically fulfil the criteria of an ideal root canal filling material. While GP used in combination with a sealer fulfils many of these requirements, one drawback of many sealers is microleakage. Microleakage can be due to differences in the thermal coefficient of expansion of the sealer and the tooth tissue, the presence of smear layer, the consistency and quantity of the endodontic sealer, the solubility of the material, or the inadequacy of the obturation. The presence of micro-spaces within the filled root canal potentially allows the penetration of tissue fluids into the root canal space. This transudate originates from blood serum. The serum undergoes degradation and diffuses into the periradicular tissue. This serum with the remaining microorganisms and their endotoxins potentiates periradicular inflammation and may ultimately contribute to treatment failure. More recently, the biological characteristics have been fulfilled with the introduction of hydraulic calcium silicate cements which are bioactive materials that appear to eliminate many of the previous concerns around microleakage.^8^

Box 1 Ideal properties of an obturating material^7^

- Easily introduced into the root canal system
- Should not shrink after being inserted
- Should be bacteriostatic or at least not encourage bacterial growth
- Should not stain tooth structure
- Should be sterile or easily and quickly sterilised immediately before insertion
- Should seal the canal laterally as well as apically
- Should be impervious to moisture
- Should be radiopaque
- Should not irritate periapical tissue
- Should be easily removed from the root canal if necessary

## Timing of obturation

Root canal treatments can be completed in one or several visits with no significant effect on outcome.^9^^,^^10^ Previously, it was thought that an interappointment dressing with non-setting calcium hydroxide paste for at least one week was beneficial, as this killed any residual microorganisms remaining after preparation and disinfection.^11^ There are certain situations where multiple visits are preferable and the clinician may opt to take this approach (Table 1).

**Table 1:** Obturation - single versus multiple visits

**Single visit**	**Multiple visits**
No pain or swelling	Patient presents with pain/swelling
No remaining pus/exudate/blood	Presence of a chronic abscess
Canals thoroughly disinfected for an adequate time	Complex treatments including perforation repair
Sufficient time to complete the procedure	Root canal filling material heavily contaminated/silver points
	Canals still wet with exudate/bleeding
	Large periapical radiolucency

It should be noted that calcium hydroxide acts by release of hydroxyl ions and therefore must be in direct contact with the bacterial cell wall to be effective. It is therefore important that the calcium hydroxide delivery system ensures that the canals are filled to length.

**Fig. 1:**
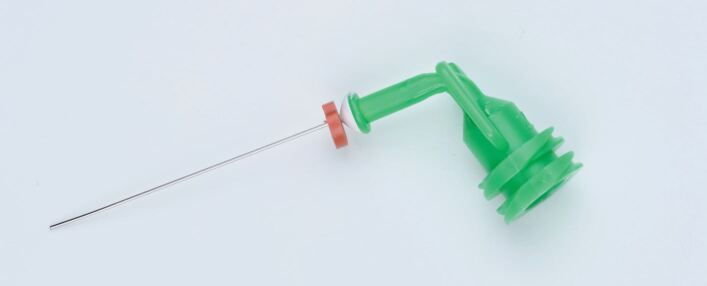
Navi tip (Ultradent, Utah, USA)

NaviTips of different lengths are effective in achieving this (Fig. 1). It has also been suggested previously that root canals should only be filled when a negative culture is obtained.^12^ Salthorn^13^ concluded, however, that the deficiencies in sampling techniques limited the predictability of using a negative culture to predict outcomes.

**Fig. 2:**
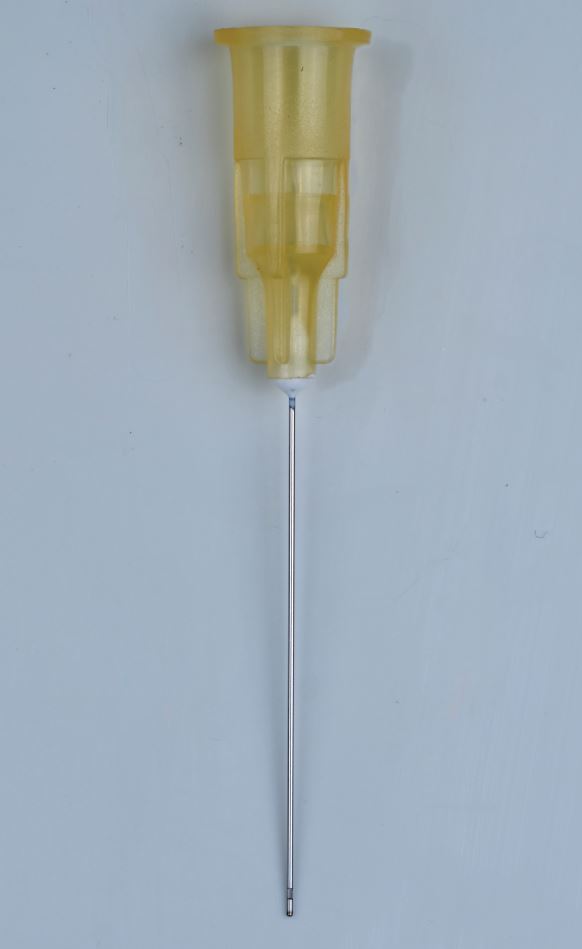
30-gauge irrigation needle (Kerr Dental, USA)

**Fig. 3:**
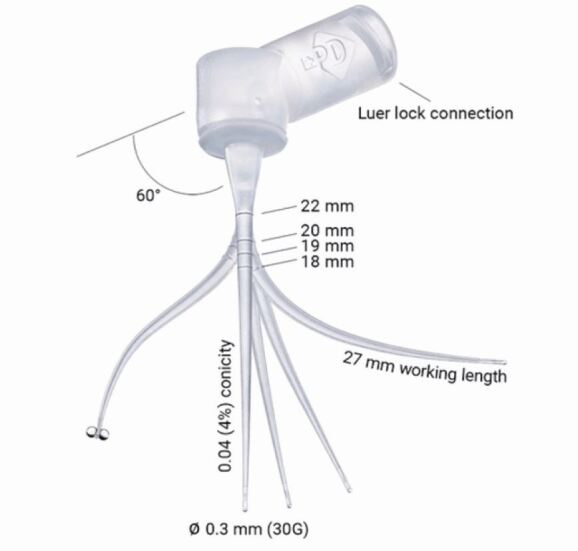
Irriflex tip (image courtesy of Quality Endodontic Distributors Limited)

With the introduction of nickel titanium files with an apical taper of at least 5%, there is more space within the apical third of the canal for exchange of irrigants and this, in combination with a smaller 30-gauge needle or Irriflex tip, will aid in destroying microorganisms in this area (Fig. 2, Fig. 3).

Completing root canal treatment in a single visit, providing the appointment time is of sufficient length, has the following advantages:

- The risk of recontamination between visits is removed
- Time and financial savings, which may be beneficial for the patient
- Less discomfort for the patient having to endure the procedure and any associated postoperative sequelae only once.

Dentists should therefore adopt a pragmatic case-by-case approach and be mindful of the small (3.17%) risk of an acute flare-up after treatment, especially in necrotic and retreatment cases, which can be more difficult to manage if the canals have been obturated in a single visit.^14^

## Obturation materials

### Gutta-percha

Dental GP (trans-polyisoprene) is the most common core material and consists of:

- Trans-polyisoprene - 19-22%
- Zine oxide - 60-75%
- Metal sulfates - 1.5-17%
- Waxes/resins - 1-4%.

GP exists in three forms, two of which are crystalline (α and β) and an amorphous form. The α-phase exhibits less shrinkage, lower viscosity and poor stability at room temperature than the β-phase. GP cones used in obturation exist in the β form. Heating to 42-49 °C, converts to α phase with the amorphous changes occurring at 68 °C.^15^ GP does not conduct heat, so when heated during warm vertical compaction procedures, the temperature at the apex is always less than the more coronal regions in contact with the heat carrier.^16^ GP shrinks on cooling^17^ and therefore is technique-dependant to ensure the required optimum seal is achieved.

**Fig. 4:**
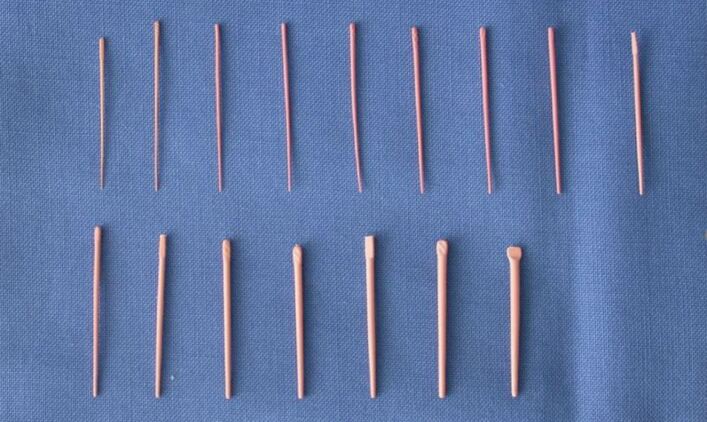
Standardised GP cones 15-140

**Fig. 5:**
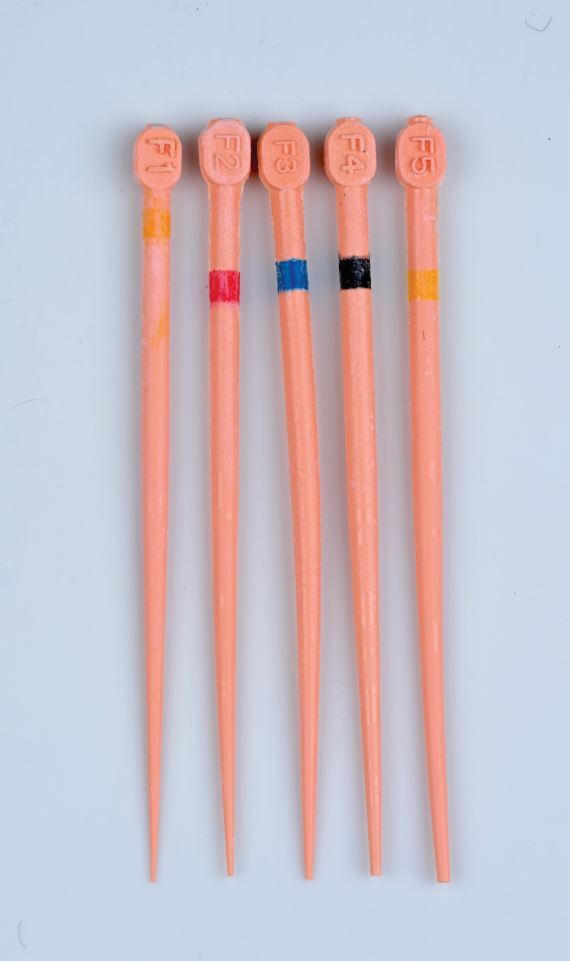
ProTaper Gold cones (Dentsply Sirona, USA)

GP cones (may also be referred to as GP points) exist in standardised and non-standardised forms. Standardised (ISO) cones have a 2% taper and tip sizes ranging from 015-140. Non-standardised cones have a tip size of 020 and range from extra-fine to extra-large. Several nickel-titanium files now come with matched cones which correlate with the taper of the file and aim to achieve a better fit within the root canal (Fig. 4, Fig. 5). The cone matching is essential in sealer-based techniques, as it minimises the volume of sealer present within the root canal, which has historically been considered as the ‘weak link' of the obturation.

Other points have been marketed such as resin points made of polycaprolactone (Resilon, Pentron Corporation, Wallingford, CT, USA); however, they have been unsuccessful due to their degradation.^18^^,^^19^^,^^20^^,^^21^^,^^22^^,^^23^^,^^24^

### Root canal sealers

A root canal sealer refers specifically to the material used in endodontic obturation to fill voids, enhance adaptation between the core material (eg GP) and the dentinal walls, and create an effective seal to prevent microleakage. Root canal sealers are used with all obturation techniques. Their chemistries and properties vary, thus it is recommended to use the sealer in such a way to optimise its specific characteristics.

The main sealer chemistries include the following:

- Zinc oxide eugenol-based
- Resin-based sealers
  - Epoxy-resin
  - Methyl methacrylate resin-based
- Hydraulic cement-based.

The zinc oxide eugenol-based sealers are based on the Grossman formulation and are presented either as a powder and liquid or two-paste systems. The powder or one of the pastes is composed of zinc oxide as the main component supplemented by staybelite resin and a number of radiopacifiers (barium sulfate and bismuth subcarbonate). The liquid and the second paste are composed of eugenol. While these sealer types are cost-effective and very popular as they are highly radiopaque so make obturations look very good on radiographs, they suffer from a number of disadvantages. The eugenol interferes with the setting of resins.^25^ This poses a substantial clinical problem as any sealer remnants in the dentinal tubules may contact with and effect the setting reaction of composite resins used to restore the tooth and also resin systems used to cement posts.^26^ Furthermore, zinc interferes with the setting of hydraulic cements.^27^ In revisions of root canal therapy, the eugenol and the zinc may also interfere with the setting of hydraulic cement and epoxy-resin based sealers, jeopardising the outcome of the revised root canal therapy. Eugenol is a known irritant and any sealer that exits beyond the apex will cause sensitisation.^28^ Zinc oxide eugenol-based sealers shrink on setting^29^^,^^30^ and thus large volumes are not recommended to be used. To this end, sealers based on zinc oxide eugenol are recommended for use with warm vertical compaction techniques where the amount of sealer used is minimal and when executed properly, the sealer does not come in contact with the heat carrier, as when heated, zinc oxide eugenol-based sealers decompose.^31^

The resin-based sealers can be based on methyl methacrylate or epoxy resin. The methacrylate-based sealer has been associated with Resilon (a resin-based material which can be used as an alternative to GP) and although it had no clinical concerns, the system was discontinued due to the failures reported.^22^^,^^23^^,^^24^ Epoxy resin-based sealers have been very popular and are considered to be a gold standard for root canal obturation. One such brand is AH Plus (Dentsply, Tulsa, OK, USA). AH Plus is presented as two pastes or a jet system with two cartridges. It is composed of paste one (diepoxide) and paste two (1-adamantane amine, N,N-dibenzyl-5-oxa-nonandaimine-1,9 and TC-diamine). Both pastes include calcium tungsten and zirconium oxide radiopacifier. The radiopacifier demonstrates the presence of the sealer and if homogenously placed, it will result in an obturation that appears well-compacted and free of voids. The setting time of AH Plus is 24-48 hours^32^ and its physical properties comply to ISO 6876:2012^33^ with adequate solubility (<3%), flow (>17 mm) and film thickness (<50 μm). AH Plus exhibits an initial expansion that stabilises with time.^29^ The sealer bonds well to dentine by micro-mechanical retention through sealer tags^34^and thus the use of a chelating agent is necessary to remove the smear layer and widening of the dentinal tubules. Epoxy resin-based sealers are good adhesives and also help bond the GP cones together. It is thus the sealer of choice for cold laterally compacted GP obturation. The antimicrobial properties are adequate compared to methacrylate-based sealers^35^ but not to hydraulic cement sealers. Thus, the final irrigating solution when using AH Plus as a sealer is recommended in the European Society of Endodontology's (ESE's) S3 guidelines^36^ to be sodium hypochlorite to maintain a low bacterial counts within the root canal space. The toxicity of AH Plus is reported to be low when compared to methacrylate resins.^37^ AH Plus cannot withstand the heat generated in warm vertical compaction^31^^,^^38^ and thus it is recommended that the sealer should not come in contact with the heat. This is relatively straightforward to control clinically as the heat carriers generally heat the GP but do not come in contact with the dentine and sealers.

Hydraulic cement sealers are materials based on the mineral trioxide aggregate (MTA) formulation and aim at enhancing the antimicrobial and biological characteristics of the materials. Because of their ability to release calcium ions, and their ability to stimulate the formation of hydroxyapatite, they have been hailed as exhibiting great promise as a ‘bioactive' root canal sealer. Only materials that set by reaction with water and interact with the environmental moisture can be classified as hydraulic cements.^39^ These materials are presented as powder and liquid which are mixed and delivered to the root canal using either a fine stainless-steel instrument (such as a DG16 probe) or a specific proprietary delivery system. Paper points may absorb the moisture from the sealer so may not be the ideal delivery system. The manufacturer's instructions state that instruments such as spiral fillers are not indicated to aid placement, as the frictional heat generated in their use can result in faster setting of the sealer.

Alternatively, the sealers are presented as a single syringe with a plastic tip. This simplifies the delivery. The sealer can only set by contact with moisture, thus there is an ongoing debate whether the moisture in the dentinal tubules is enough and whether it reaches the sealer. Most certainly, with these sealer types, the removal of the smear layer by chelating agents is necessary, as this removes the tubule occlusion. In clinical practice, these sealers are collectively referred to as bioceramics. This results in a degree of confusion as the chemistry of the sealer varies and the exact composition is not captured in the name.

Most of the hydraulic cement sealers are composed of the hydraulic cement, a radiopacifier, additives to enhance the sealer properties (flow and film thickness) and a vehicle. The quantity and chemistry of the hydraulic cement component is highly variable, with some sealers reported to have less than 15% of an active component and being mostly composed of radiopacifier.^40^ The cement chemistry is also assumed to be tricalcium silicate as this is the MTA composition. There are various hydraulic cements and only the tricalcium silicates interact with water to produce calcium hydroxide and a calcium silicate hydrate.^41^ The radiopacifiers vary in quantity^40^ and chemistry with zirconium oxide is most frequently used, as opposed to the bismuth oxide in the original formulation of MTA that was shown to discolour teeth and thus should be avoided at all costs.^41^

Hydraulic cement sealers do not shrink unless they are desiccated;^42^ however, they have been reported to be highly soluble. The high solubility may be due to inappropriate standards^33^ to test against, since the materials interact with the environment to precipitates on the surface and this skews the gravimetric method used to assess the solubility.^43^ The calcium hydroxide produced as a by-product of the interaction of tricalcium silicate with water is responsible for the antimicrobial and biological properties of the sealers.^44^ The sealers interact with dentine and result in alkaline etch with diffusion of mineral from the sealer into the dentine, resulting in a mineral infiltration zone and also sealer tags.^34^

These sealer types have positively changed the principles of root canal obturation from quality of fill to a more biological approach with active elimination of microbial contamination. Although the sealers are not toxic and enhance bone differentiation, it is still not advisable to have extrusions beyond the apex due to the high alkalinity, which may result in soft tissue burns and damage to nerves in close proximity to the root apices. Hydraulic cements are best used in sealer-based techniques, such as single-cone obturation, due to their biological properties and also their interaction with the dentine. The materials that are mixed with water should not be heated as the water evaporation will lead to deterioration of sealer physical properties.^38^ Single-syringe sealers use alternative vehicles so do not suffer this shortcoming,

## Obturation techniques

To improve success rates, obturation should be within 2 mm of the root apex and contain no voids.^45^ Ideally, the GP should end at the cemento-dentinal junction which can range from 0-3 mm from the radiographic apex.^46^ Electronic apex locators are recommended to determine the position of the apical constriction, as this is more accurate compared to taking a working length (WL) radiograph.^47^

## Cone-fit radiograph

It is useful following to take a cone-fit radiograph on completion of root canal preparation and disinfection. This has the following benefits:

- Ensures accurate cone placement to WL before obturation
- Allows assessment of cone fit, apically
- Identifies merging canals, obstructions or ledges
- Gives a visual recording of cone placement.

### Technique

Following final irrigation and agitation (or activation), the canals are left with irrigant *in situ.* Cones are selected to match with the final shaping file. These are disinfected by placing a hypochlorite solution for one minute. Each cone is measured to the correct WL and carefully inserted into the canal without bending. When WL is reached, the cone is checked for apical fit. It should feel ‘snug' and it should not be possible to move it beyond WL and into the periapical tissues. If the cone fit is not perfect, modifications can be made to either the cone size or preparation before the final obturation.

When two canals merge, after the placement of the first cone, it will not be possible to seat the second cone to the correct length. An easy way to manage this is to measure how many millimetres the second cone is short of the WL and cut this from the tip of the cone.

Once the cone-fit radiograph has been checked, the canals can be dried with sterile paper points ready for obturation.

If new cones are selected for obturation, these too should be disinfected in sodium hypochlorite and left to dry on sterile gauze. Following the cone-fit radiograph and before obturation, recent research has suggested that the clinician should adopt an enhanced infection protocol, including changing gloves to reduce any unnecessary microbial contamination.^48^

Obturation techniques can be broadly divided into cold or warm techniques, as below.

### Cold techniques

1. Cold lateral compaction
2. Single cone with a calcium silicate sealer.

#### Cold lateral compaction

**Fig. 6:**
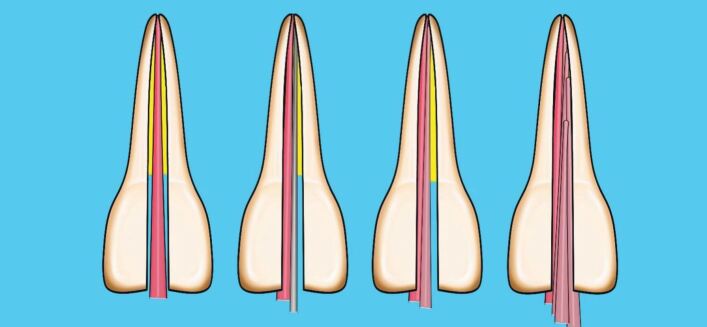
Diagram of cold lateral compaction technique

**Fig. 7:**
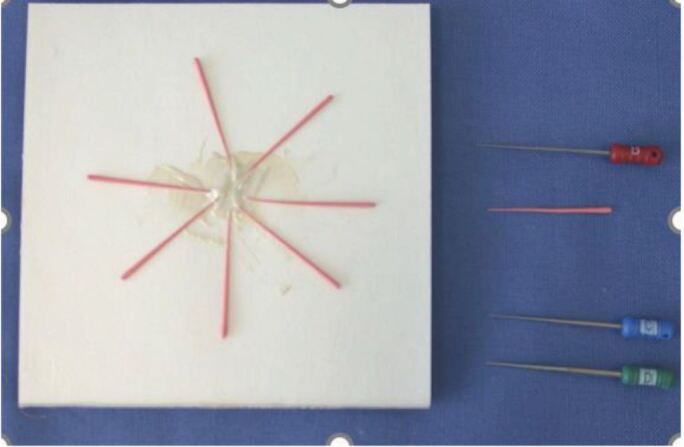
Sealer, vcone and spreader

This involves placing a cone, lightly coated in sealer, to length and then using a metal spreader to compact it against the canal wall. This creates space to then insert a smaller GP cone, known as an accessory cone, alongside the master cone. This process is repeated until no space remains. It is also known as cold lateral condensation; however, this term does not accurately describe the procedure and should no longer be used. This technique relies on the sealer to fill the spaces between the GP cones (Fig. 6, Fig. 7). A step-by-step guide of the technique is shown in Table 2.

**Table 2:** Step-by step guide for cold lateral compaction

Step 1	Have all materials ready to hand including the master cone, accessory cones with matching finger spreader and sealer (Fig. 7). Size B works in most cases and should have a rubber stop measured at 2 mm short of WL. Sealer dispensed on a paper pad, a ruler and locking tweezers
Step 2	Take the master cone and measure to correct WL. Coat lightly in sealer and place in canal using a gentle pumping motion until it reaches WL
Step 3	Take the size B finger spreader and place it alongside the cone, 1-2 mm short of WL, maintaining light apical pressure for ten seconds to allow the cone to be compacted against the canal wall
Step 4	Slowly withdraw the spreader, ensuring that you do not dislodge the cone
Step 5	The space created will close quickly, so without wasting time, take an accessory cone, coated lightly in sealer, and place into the channel created
Step 6	Continue until spreader cannot penetrate further than 3-4 mm from canal orifice
Step 7	Repeat for other canals
Step 8	The excess GP can be removed using a heated flat plastic, ensuring no traces remain on the pulpal floor. Sealer can be removed from the pulp chamber by cleaning with alcohol on a microbrush

##### Disadvantages

- The apical seal is wholly dependent on sealer, as there is no heat applied to the GP apically
- Higher risk of creating vertical root fractures due to the forces generated during compaction of the obturation material.^49^

#### Single cone with a calcium silicate sealer (sealer-based technique)

**Fig. 8:**
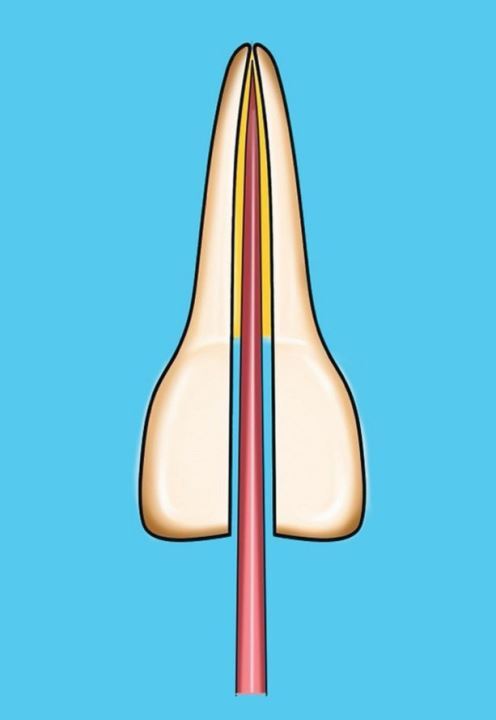
Diagram of single cone calcium silicate sealer technique

In this technique, a cone matched to the size of the final shaping file is selected, and once the fit has been checked as above, the cone is disinfected and dried. The coronal two-thirds of the root canal is gently filled with hydraulic calcium silicate sealer using the proprietary small diameter tip and the cone, measured to length, placed into the canal using a gentle pumping motion. This will carry the sealer down into the apical third of the canal (Fig. 8).

The sealer, due to its high flowability, can be seen to penetrate well into lateral canals and apical anatomy on post-treatment radiographs, ensuring a good seal; however, it can also extend into the periapical tissues. The technique is favoured by nickel-titanium file systems that produce a much more conservative canal preparation.

The single-cone obturation technique is sealer-based, as a single GP cone is used and the rest of the space is filled with sealer. The sealer properties are key and the use of hydraulic cement sealers is recommended. This technique is very popular due to its simplicity and lack of specialised armamentarium.^50^ Since hydraulic cements interact with the environment they are placed in, the final irrigating solution is important. Chlorhexidine should be avoided as a final irrigant^51^ and the best irrigation protocol has been found to be sodium hypochlorite followed by a calcium chelator, such as ethylenediaminetetraacetic acid, with a final rinse of water. Solutions reducing the pH should be avoided as there will be a tendency to bacterial recolonisation.^11^ The water leaves the dentine adequately hydrated and the calcium chelation removes the smear layer, enabling better adaptation of the sealer with the dentine.

### Warm techniques

1. Warm vertical compaction
2. Continuous wave obturation
3. Carrier-based
4. Thermomechanical compaction.

#### Warm vertical compaction

First described by Schilder in 1967,^52^ to create a homogeneous mass of GP, closely adapted to the canal wall and sealing lateral canals, isthmuses and the apical delta. This should prevent any residual microorganisms and their by-products from gaining access to the periapical tissues.

A tapered master cone coated in sealer is fitted 0.5-2 mm short of the working length and checked for ‘tug back', which is better defined as resistance to displacement. The coronal portion is removed using heat and a cold plugger is used to compact the softened GP apically to complete the apical seal. A small pellet of GP is placed in the canal and heat applied. A cold plugger is then used to compact and move the softened GP apically. This is repeated in the middle and coronal thirds of the canal.

#### Continuous wave obturation

This has become a popular variation of warm vertical compaction, described by Buchanan in 1996,^53^ and is used by many endodontists today. It requires a tapered preparation and has two components, known as downpack and backfill. The thermoplastic GP will fill lateral canals, isthmuses and internal resorption defects.

##### Downpack

**Fig. 9:**
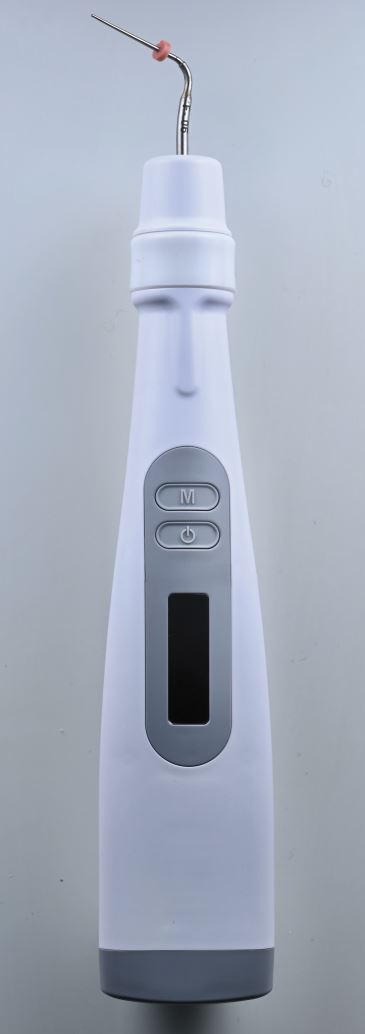
Elements IC Downpack Unit (Kerr Dental, USA)

**Fig. 10:**
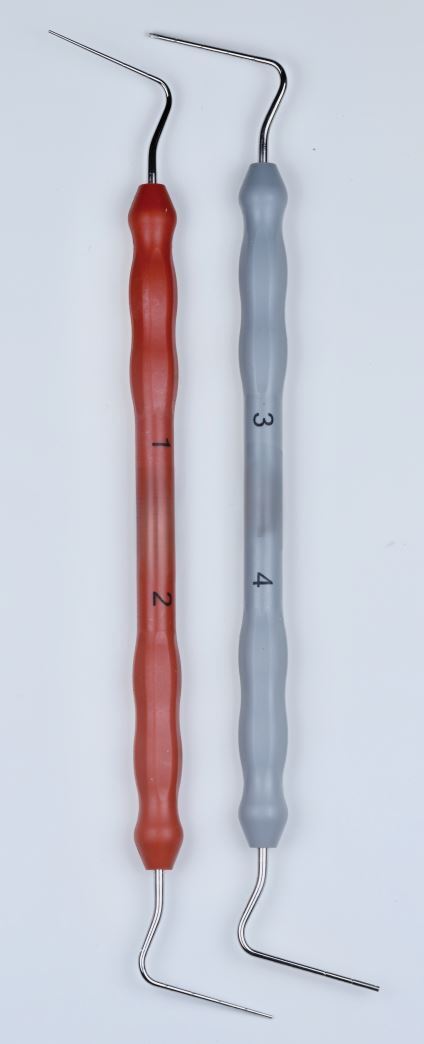
Dr Machtou pluggers (Dentsply, USA)

A heated plugger is selected to fit snugly within the root canal, 5-7 mm short of the WL. Once the master cone has been checked for fit, it is lightly coated in sealer and inserted into the canal. The heated plugger, set at 200 °C, is gently pushed through the master cone to the predetermined length, taking no more than three seconds. The heat is removed and light apical pressure maintained for ten seconds to counteract any space created by shrinkage on cooling. The heat is again activated in a short burst of one second and the plugger withdrawn. This will remove the coronal GP. A flat plugger is then used to compact the apical portion of GP (Fig. 9, Fig. 10).

##### Backfill

**Fig. 11:**
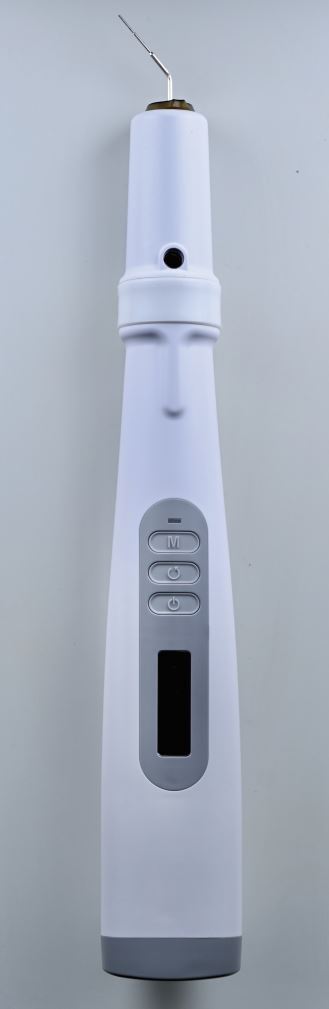
Elements IC Backfill Unit (Kerr Dental, USA)

Many devices are used for this technique. They have different gauge needles depending on the diameter of the canal. One should be selected that reaches the apical GP, 5-7 mm short of the WL (Fig. 11). It is important to note that although that temperature on the readout indicates 200 °C, the temperatures of the tips are all below 100 °C.^31^ The temperature of conversion of GP is 65 °C,^15^^,^^16^^,^^17^ thus higher temperatures are not necessary and will risk in damaging the adjacent periodontal ligament space and bone.

##### Step-by-step guide

**Fig. 12:**
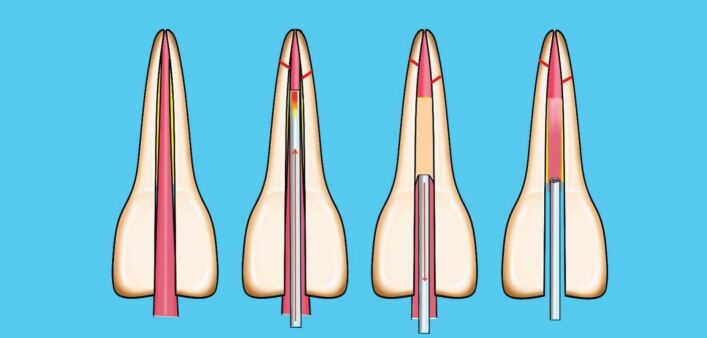
Diagram of technique, continuous wave compaction

The choice of sealer for continuous wave obturation has always been zinc oxide eugenol-based sealers. However, since all sealers decompose in the presence of heat, it is important to avoid heating the sealers during the obturation. The coronal part of the canal should be coated lightly with sealer and downpacked as described above. During the backfill phase, place the needle tip until it contacts the apical GP. Maintain in this position for a few seconds before slowly extruding the injectable GP and at the same time, slowly withdraw the needle from the canal. Once the level of the canal orifice has been reached, remove the needle and compact with a flat plugger (Fig. 12).

#### Carrier-based, thermoplastic techniques

**Fig. 13:**
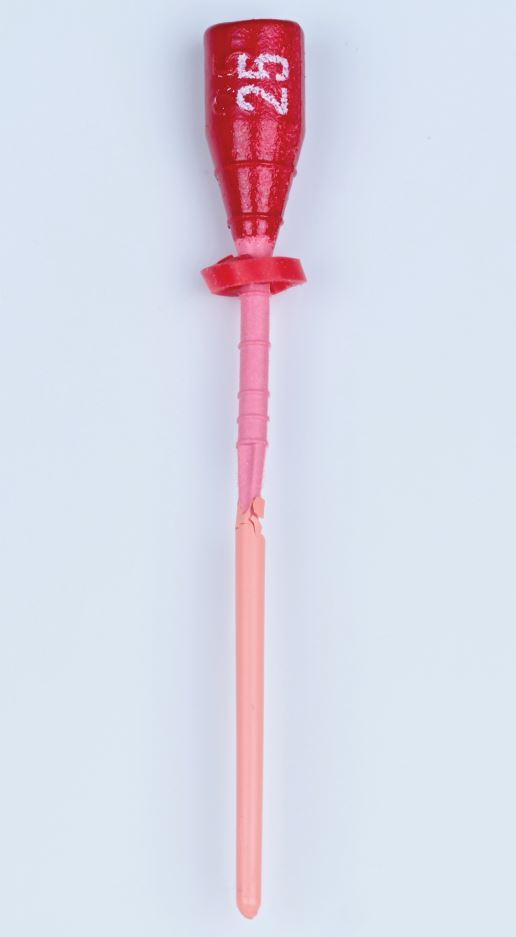
Guttacore (Dentsply Sirona, USA)

The original technique, as described by Johnston in 1978,^54^ used a metal core coated in solid GP that was heated and placed in the root canal. Johnston realised the challenges of this technique with regard to re-treatment in particular and designed a replacement using a solid plastic core with alpha GP in the 1990s. The plastic gave the obturator more flexibility; however, removal of the plastic core is challenging, especially in narrow and/or curved canals. The GP could also be inadvertently stripped off the obturator on placement, leaving the apical part of the canal filled only with the plastic core. A crosslinked core material made from GP, known as GuttaCore (Fig. 13), has been introduced more recently to facilitate easier removal in retreatment cases. It is available in sizes specific to the range of Dentsply Sirona file systems.

##### Step-by-step guide

- As the obturator cannot be tried into the canal, it is recommended to use a verifier which matches the preparation, or ensure the shaping file can fit passively in the canal to the correct working length
- The depth rings on the handle of the obturator are used to set the WL using the silicone stop
- While the obturator is heating in the dedicated oven set at 135 °C, a small amount of sealer is placed in the coronal part of the canal using a DG16 or paper point
- The obturator is removed from the oven and placed in the canal to WL, taking no longer than ten seconds and without rotating or twisting
- The handle is removed using an excavator or heated instrument and coronal GP, compacted using a flat plugger
- Any material left within the pulp chamber can be removed with a heated instrument.

As with all thermoplastic techniques, there is a risk of extruding excess material apically. To avoid this, minimal sealer should be used,and the obturator ideally seated 0.5 mm less than WL.

#### Thermomechanical compaction

**Fig. 14:**
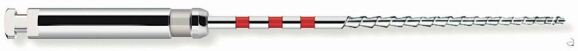
McSpadden condenser (image courtesy of Quality Endodontic Distributors Limited)

A McSpadden compactor (Fig. 14), which is based on a reverse Hedstrom file, is placed in the canal, alongside the master cone coated in sealer and driven at 5,000-10,000 rpm to within 3-4 mm from the WL. The frictional heat generated warms, plasticises and compacts the GP towards the canal walls. The technique is not suitable for narrow curved canals. It also has disadvantages in that the compactors can break within the canal and the softened GP can easily be extruded into the periapical tissues. There is also some concern regarding the elevated temperature created within the root canal.^55^

This technique, although used historically by some endodontists, is rarely used as the compactors are not now commercially available.

## Complex cases and complications

### Wide/open apex

**Fig. 15:**
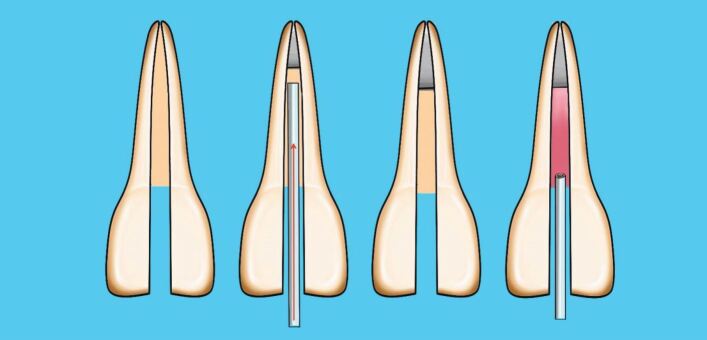
Diagram of technique for apical plug placement in wide apex

Canals can have wide apices, either by becoming non-vital prematurely or by destruction of the apical anatomy by inflammatory root resorption. Historically, apexification using calcium hydroxide over several months would have been used to create a barrier to allow obturation without extrusion.^56^ Since the introduction of mineral trioxide aggregate, hydraulic calcium silicate cements have been used to place an immediate apical barrier and are indicated in cases where the apical opening is gauged at 0.70 mm or greater (Fig. 15). A step-by-step guide is given in Table 3.

**Table 3:** Step-by-step guide for management of complex cases with open apices

Step 1	Magnification, preferably using a microscope, is recommended for this procedure
Step 2	The canal is dried using large paper points, ensuring these are measured just short of WL so the periapical tissues are not disturbed which can cause bleeding to enter the canal
Step 3	A specific carrier is used to dispense a small amount of material apically, trying to keep it from attaching to the canal walls
Step 4	A flat plugger with a rubber stop 0.5 mm shorter than WL is used to gently tap the material into place
Step 5	Further increments are added and gently packed into place until 4-5 mm has been achieved
Step 6	A radiograph is advisable at this point to ensure good adaption of the material apically
Step 7	The canal is then backfilled with thermoplastic GP, ensuring there are no voids

### Internal inflammatory root resorption

This is rare, with a prevalence of 0.01-1% and an aetiology and pathogenesis that is poorly understood. It is often confused with cervical invasive resorption; however, unlike cervical resorption and external inflammatory root resorption, its progression is halted once the whole pulp becomes necrotic. Often asymptomatic, radiographically it presents with a round or oval and symmetrical widening of the root canal space and the original canal shape is lost.

Cleaning of the resultant root canal space can be challenging and relies heavily on chemical disinfection obturation which requires a thermoplastic technique to fill the irregular shape. Depending on the location, a master cone can be sealed apical to the lesion and injectable GP used to fill the remaining canal. It has been suggested that if a perforation is present, MTA can be used; however, this can be challenging to adapt to such a shape. It may also be necessary to place fibre posts where the root dentine has become very thin as GP will not confer any structural reinforcement.

### Overfill, overextension

**Fig. 16:**
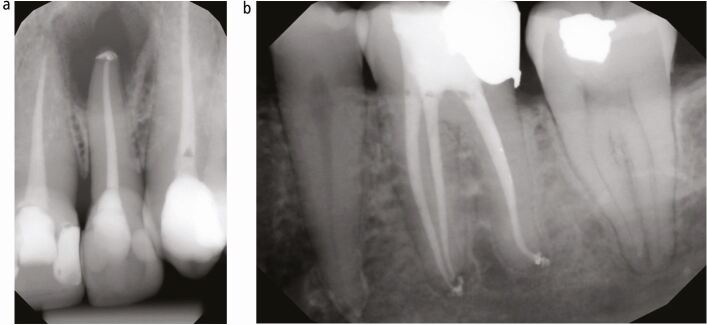
a, b) Obturation with apical puffs of calcium silicate sealer

GP is a relatively inert material that does not support bacterial growth and has been shown to be well-tolerated by the periapical tissues.^57^ Ideally, root canal filling material should fill the canal space to the apical constriction without extruding into the periapical tissues. Overfilling can occur because of a lack or loss of the apical constriction due to inflammatory resorption, an immature root apex, over-instrumentation, errors with selection and placement of the master cone, and thermoplastic techniques. Today, with the wide use of calcium silicate sealers, it is common to see ‘puffs' of sealer apical to the root apex (Fig. 16). Due to their hydrophilic nature, the containment of hydraulic cements within the canal is difficult. Although the puffs indicate that all the root canal anatomy has been prepared and filled, overfilling should be avoided due to the alkalinity of the sealers, regardless of the bioactive nature of the hydraulic cements.

Overextension refers to GP also extruded beyond the root apex but in this case, the GP does not completely fill the canal system and residual microorganisms may have a negative impact on biological outcomes.

### Underfill, underextension

Underfilling can occur due to poorly fitted GP points where although the root canal has been adequately prepared, the obturation is short of the apex. This usually has less impact on the outcome of root canal therapy than overfilling. If under-extension occurs due to an inadequately prepared canal space, debris generated during root canal instrumentation will be present in the apical third, impeding the adequate obturation to the apex.

## Discussion and conclusions

While various materials and techniques are available, the latest S3-level guidelines for managing pulpal and periapical disease^36^ indicate that clinical outcomes remain consistent regardless of the sealer or obturation technique used.

Ultimately, the success of obturation depends on various factors throughout the entire root canal treatment process. The techniques and approaches outlined in this article serve as a guide for clinicians, reinforcing best practice, to aid in selecting appropriate obturation methods based on their clinical judgement, experience and individual case requirements.

The guidelines also highlight the importance of aseptic techniques, proper training and regular re-evaluations during and after treatment to ensure long-term success.

In summary, the ESE's S3-level guidelines advocate for a holistic approach to endodontic treatment, where the entire process - from diagnosis to obturation - is optimised to achieve the best possible patient outcomes.

As advancements in knowledge and technique continues, staying informed remains essential to achieving optimal patient outcomes.

## References

[1] Ray H A, Trope M. Periapical status of endodontically treated teeth in relation to the technical quality of the root filling and the coronal restoration. *Int Endod J* 1995; **28:** 12-18. 10.1111/j.1365-2591.1995.tb00150.x 7642323

[2] Grossman L I. A brief history of endodontics. *J Endod* 1982; **8:** 536-540.

[3] Peters O A, Schönenberger K, Laib A. Effects of four Ni-Ti preparation techniques on root canal geometry assessed by micro computed tomography. *Int Endod J* 2001; **34:** 221-230. 10.1046/j.1365-2591.2001.00373.x 12193268

[4] Boutsioukis C, Arias-Moliz M T. Present status and future directions - irrigants and irrigation methods. *Int Endod J* 2022; **55:** 588-612. 10.1111/iej.13739 PMC9321999 35338652

[5] Boutsioukis C, Nova P G. Syringe irrigation in minimally shaped root canals using 3 endodontic needles: a computational fluid dynamics study. *J Endod* 2021; **47:** 1487-1495. 10.1016/j.joen.2021.06.001 34118256

[6] Kirsten H M, Moorer W R. Particles and molecules in endodontic leakage. *Int Endod J* 1989; **22:** 118-124. 10.1111/j.1365-2591.1989.tb00909.x 2634619

[7] Grossman L I, Oliet S, Del Rio C E. *Endodontic practice*. Philadelphia: Lea & Febiger, 1988.

[8] Vula V, Stavileci M, Ajeti N, Vula V, Kuçi A, Meqa K. Evaluation of apical leakage after root canal obturation with glass ionomer, resin, and zinc oxide eugenol sealers combined with thermafil. *Med Sci Monit Basic Res* 2022; DOI: 10.12659/MSMBR.936675. 10.12659/MSMBR.936675 PMC9208302 35771493

[9] Peters L B, Wesselink P R. Periapical healing of endodontically treated teeth in one and two visits obturated in the presence or absence of detectable microorganisms. *Int Endod J* 2002; **35:** 660-667. 10.1046/j.1365-2591.2002.00541.x 12196219

[10] Penesis V A, Fitzgerald P I, Fayad M I, Wenckus C S, BeGole E A, Johnson B R. Outcome of one-visit and two-visit endodontic treatment of necrotic teeth with apical periodontitis: a randomized controlled trial with one-year evaluation. *J Endod* 2008; **34:** 251-257. 10.1016/j.joen.2007.12.015 18291270

[11] Arias-Moliz M, Camilleri J. The effect of the final irrigant on the antimicrobial activity of root canal sealers. *J Dent* 2016; **52:** 30-36. 10.1016/j.jdent.2016.06.008 27377571

[12] Sjögren U, Figdor D, Persson S, Sundqvist G. Influence of infection at the time of root filling on the outcome of endodontic treatment of teeth with apical periodontitis. *Int Endod J* 1997; **30:** 297-306. 10.1046/j.1365-2591.1997.00092.x 9477818

[13] Salthorn C, Parashos P, Messer H H. How useful is root canal culturing in predicting treatment outcome? *J Endod* 2007; **33:** 220-225. 10.1016/j.joen.2006.11.006 17320700

[14] Walton R, Fouad A. Endodontic interappointment flare-ups: a prospective study of incidence and related factors. *J Endod* 1992; **18:** 172-177. 10.1016/S0099-2399(06)81413-5 1402571

[15] Schilder H, Goodman A, Aldrich W. The thermomechanical properties of gutta-percha. 3. Determination of phase transition temperatures for gutta-percha. *Oral Surg Oral Med Oral Pathol* 1974; **38:** 109-114. 10.1016/0030-4220(74)90321-1 4525950

[16] Goodman A, Schilder H, Aldrich W. The thermomechanical properties of gutta-percha. Part IV. A thermal profile of the warm gutta-percha packing procedure. *Oral Surg Oral Med Oral Pathol* 1981; **51:** 544-551. 10.1016/0030-4220(81)90017-7 6941146

[17] Schilder H, Goodman A, Aldrich W. The thermomechanical properties of gutta-percha Part V. Volume changes in bulk gutta-percha as a function of temperature and its relationship to molecular phase tranformation. *Oral Surg Oral Med Oral Pathol* 1985; **59:** 285-296. 10.1016/0030-4220(85)90169-0 3856822

[18] Hiraishi N, Yau J Y, Loushine R J *et al*. Susceptibility of a polycaprolactone-based root canal-filling material to degradation. III. Turbidimetric evaluation of enzymatic hydrolysis. *J Endod* 2007; **33:** 952-956. 10.1016/j.joen.2007.05.004 17878081

[19] Tay F R, Pashley D H, Williams M C *et al*. Susceptibility of a polycaprolactone-based root canal filling material to degradation. I. Alkaline hydrolysis. *J Endod* 2005; **31:** 593-598. 10.1097/01.don.0000152301.72828.61 16044043

[20] Tay F R, Pashley D H, Yiu C K *et al*. Susceptibility of a polycaprolactone-based root canal filling material to degradation. II. Gravimetric evaluation of enzymatic hydrolysis. *J Endod* 2005; **31:** 737-741. 10.1097/01.don.0000155225.40794.79 16186753

[21] Tay F R, Pashley D H, Loushine R J *et al*. Susceptibility of a polycaprolactone-based root canal filling material to degradation. Evidence of biodegradation from a simulated field test. *Am J Dent* 2007; **20:** 365-369. 18269126

[22] Payne L A, Tawil P Z, Phillips C, Fouad A F. Resilon: assessment of degraded filling material in nonhealed cases. *J Endod* 2019; **45:** 691-695. 10.1016/j.joen.2019.02.019 31005333

[23] Barborka B J, Woodmansey K F, Glickman G N, Schneiderman E, He J. Long-term clinical outcome of teeth obturated with Resilon. *J Endod* 2017; **43:** 556-560. 10.1016/j.joen.2016.12.005 28342476

[24] Strange K A, Tawil P Z, Phillips C, Walia H D, Fouad A F. Long-term outcomes of endodontic treatment performed with resilon/epiphany. *J Endod* 2019; **45:** 507-512. 10.1016/j.joen.2019.01.019 30905575

[25] Garcia I M, Leitune V C, Ibrahim M S *et al*. Determining the effects of eugenol on the bond strength of resin-based restorative materials to dentin: a meta-analysis of the literature. *Appl Sci* 2020; **10:** 1070.

[26] Cecchin D, Farina A P, Souza M A, Carlini-Júnior B, Ferraz C C R. Effect of root canal sealers on bond strength of fibreglass posts cemented with self-adhesive resin cements. *Int Endod J* 2011; **44:** 314-320. 10.1111/j.1365-2591.2010.01831.x 21219360

[27] Camilleri J. Scanning electron microscopic evaluation of the material interface of adjacent layers of dental materials. *Dent Mater* 2011; **27:** 870-878. 10.1016/j.dental.2011.04.013 21565396

[28] Scelza M Z, Linhares A B, Da Silva L E, Granjeiro J M, Alves G G. A multiparametric assay to compare the cytotoxicity of endodontic sealers with primary human osteoblasts. *Int Endod J* 2012; **45:** 12-18. 10.1111/j.1365-2591.2011.01941.x 21902702

[29] Ørstavik D, Nordahl I, Tibballs J E. Dimensional change following setting of root canal sealer materials. *Dent Mater* 2001; **17:** 512-519. 10.1016/s0109-5641(01)00011-2 11567689

[30] Hammad M, Qualtrough A, Silikas N. Extended setting shrinkage behavior of endodontic sealers. *J Endod* 2008; **34:** 90-93. 10.1016/j.joen.2007.10.014 18155502

[31] Atmeh A R, Hadis M, Camilleri J. Real-time chemical analysis of root filling materials with heating: guidelines for safe temperature levels. *Int Endod J* 2020; **53:** 698-708. 10.1111/iej.13269 31955442

[32] Zhou H-M, Shen Y, Zheng W, Li L, Zheng Y-F, Haapasalo M. Physical properties of 5 root canal sealers. *J Endod* 2013; **39:** 1281-1286. 10.1016/j.joen.2013.06.012 24041392

[33] International Organization for Standardization. ISO 6876:2012. Dentistry - root canal sealing materials. 2012. Available at https://www.iso.org/standard/45117.html (accessed March 2025).

[34] Viapiana R, Guerreiro-Tanomaru J, Tanomaru-Filho M, Camilleri J. Interface of dentine to root canal sealers. *J Dent* 2014; **42:** 336-350. 10.1016/j.jdent.2013.11.013 24287256

[35] Prestegaard H, Portenier I, Ørstavik D, Kayaoglu G, Haapasalo M, Endal U. Antibacterial activity of various root canal sealers and root-end filling materials in dentin blocks infected *ex vivo* with Enterococcus faecalis. *Acta Odontol Scand* 2014; **72:** 970-976. 10.3109/00016357.2014.931462 25005627

[36] Duncan H F, Kirkevang L-L, Peters O A *et al*. Treatment of pulpal and apical disease: the European Society of Endodontology (ESE) S3-level clinical practice guideline. *Int Endod J* 2023; **56:** 238-295. 10.1111/iej.13974 37772327

[37] Lodienė G, Morisbak E, Bruzell E, Ørstavik D. Toxicity evaluation of root canal sealers *in vitro*. *Int Endod J* 2008; **41:** 72-77. 10.1111/j.1365-2591.2007.01321.x 17931390

[38] Camilleri J. Sealers and warm gutta-percha obturation techniques. *J Endod* 2015; **41:** 72-78. 10.1016/j.joen.2014.06.007 25115660

[39] Camilleri J. Classification of hydraulic cements used in dentistry. *Front Dent Med* 2020; **1:** 9. 10.1111/iej.70180 PMC13462355 42108712

[40] Cardinali F, Camilleri J. A critical review of the material properties guiding the clinician's choice of root canal sealers. *Clin Oral Investig* 2023; **27:** 4147-4155. 10.1007/s00784-023-05140-w PMC10415471 37460901

[41] Camilleri J. Color stability of white mineral trioxide aggregate in contact with hypochlorite solution. *J Endod* 2014; **40:** 436-440. 10.1016/j.joen.2013.09.040 24565667

[42] Camilleri J, Mallia B. Evaluation of the dimensional changes of mineral trioxide aggregate sealer. *Int Endod J* 2011; **44:** 416-424. 10.1111/j.1365-2591.2010.01844.x 21255041

[43] Kebudi Benezra M, Schembri Wismayer P, Camilleri J. Influence of environment on testing of hydraulic sealers. *Sci Rep* 2017; **7:** 17927. 10.1038/s41598-017-17280-7 PMC5738414 29263328

[44] Camilleri J, Atmeh A, Li X, Meschi N. Present status and future directions: hydraulic materials for endodontic use. *Int Endod J* 2022; **55:** 710-777. 10.1111/iej.13709 PMC9314068 35167119

[45] Ng Y-L, Mann V, Rahbaran S, Lewsey J, Gulabivala K. Outcome of primary root canal treatment: systematic review of the literature - part 2. Influence of clinical factors. *Int Endod J* 2008; **41:** 6-31. 10.1111/j.1365-2591.2007.01323.x 17931388

[46] Schaeffer M A, White R R, Walton R E. Determining the optimal obturation length: a meta-analysis of literature. *J Endod* 2005; **31:** 271-274. 10.1097/01.don.0000140585.52178.78 15793382

[47] Martins J N, Marques D, Mata A, Caramês J. Clinical efficacy of electronic apex locators: systematic review. *J Endod* 2014; **40:** 759-777. 10.1016/j.joen.2014.03.011 24862702

[48] Patel S, Puri T, Mannocci F, Bakhsh A A. The outcome of endodontic treatment using an enhanced infection protocol in specialist practice. *Br Dent J* 2022; **232:** 805-811. 10.1038/s41415-022-4339-y 35689064

[49] Gimlin D R, Parr C H, Aguirre-Ramirez G. A comparison of stresses produced during lateral and vertical condensation using engineering models. *J Endod* 1986; **12:** 235-241. 10.1016/S0099-2399(86)80254-0 3461109

[50] Guivarc'h M, Jeanneau C, Giraud T *et al*. An international survey on the use of calcium silicate-based sealers in non-surgical endodontic treatment. *Clin Oral Investig* 2020; **24:** 417-424. 10.1007/s00784-019-02920-1 31104112

[51] Kapralos V, Valen H, Koutroulis A, Camilleri J, Ørstavik D, Sunde P T. The dentine-sealer interface: modulation of antimicrobial effects by irrigation. *Int Endod J* 2022; **55:** 544-560. 10.1111/iej.13692 PMC9303667 35080277

[52] Schilder H. Filling root canals in three dimensions. *Dent Clin North Am* 1967; **11:** 723-744. 5262492

[53] Buchanan L S. The continuous wave of condensation technique: a convergence of conceptual and procedural advances in obturation. *Dent Today* 1994; **13:** 80-85. 9540580

[54] Johnson W B. A new gutta-percha technique. *J Endod* 1978; **4:** 184-188. 10.1016/S0099-2399(78)80173-3 284089

[55] Saunders E M. *In vivo* findings associated with heat generation during thermomechanical compaction of gutta-percha. 2. Histological response to temperature elevation on the external surface of the root. *Int Endod J* 1990; **23:** 268-274. 10.1111/j.1365-2591.1990.tb00860.x 2098344

[56] Frank A L. Therapy for the divergent pulpless tooth by continued apical formation. *J Am Dent Assoc* 1966; **72:** 87-93. 10.14219/jada.archive.1966.0017 5215726

[57] Tavares T, Scares I J, Silveira N L. Reaction of rat subcutaneous tissue to implants of gutta-percha for endodontic use. *Endod Dent Traumatol* 1994; **10:** 174-178. 10.1111/j.1600-9657.1994.tb00682.x 7995248

